# Scientists Want More Children

**DOI:** 10.1371/journal.pone.0022590

**Published:** 2011-08-05

**Authors:** Elaine Howard Ecklund, Anne E. Lincoln

**Affiliations:** 1 Department of Sociology, Rice University, Houston, Texas, United States of America; 2 Department of Sociology, Southern Methodist University, Dallas, Texas, United States of America; University of Maribor, Slovenia

## Abstract

Scholars partly attribute the low number of women in academic science to the impact of the science career on family life. Yet, the picture of how men and women in science – at different points in the career trajectory – compare in their perceptions of this impact is incomplete. In particular, we know little about the perceptions and experiences of junior and senior scientists at top universities, institutions that have a disproportionate influence on science, science policy, and the next generation of scientists. Here we show that having fewer children than wished as a result of the science career affects the life satisfaction of science faculty and indirectly affects career satisfaction, and that young scientists (graduate students and postdoctoral fellows) who have had fewer children than wished are more likely to plan to exit science entirely. We also show that the impact of science on family life is not just a woman's problem; the effect on life satisfaction of having fewer children than desired is more pronounced for male than female faculty, with life satisfaction strongly related to career satisfaction. And, in contrast to other research, gender differences among graduate students and postdoctoral fellows disappear. Family factors impede talented young scientists of both sexes from persisting to research positions in academic science. In an era when the global competitiveness of US science is at risk, it is concerning that a significant proportion of men and women trained in the select few spots available at top US research universities are considering leaving science and that such desires to leave are related to the impact of the science career on family life. Results from our study may inform university family leave policies for science departments as well as mentoring programs in the sciences.

## Introduction

Qualified and interested women are still kept out and drop out of academic science [1], [2]. Concern about the impact a science career will have on family life is one reason. Researchers have initially focused on explaining why having children seems to have a disproportionate impact on women's academic lives [3], [4]. For example, researchers argue that mothers may be particularly less likely to put in the work hours necessary to maintain a high-level science career [5], [6]. And research shows that family concerns weigh on male scientists as well, since they are often the primary breadwinners for their families [2].

Research that examines the impact of the scientific career on family life has several limitations. Single-institution or university-system case studies limit generalizability [2], [6]. Low response rates are another common weakness [7]. Many studies on this topic have achieved response rates far less than even 50 percent, raising serious concerns about non-respondent bias [2], [3], [6]. Other studies are descriptive, rather than adjusting for differences among respondents [6]. Finally, researchers know little about how faculty experiences at leading institutions compare to the experiences or *projected* experiences of scientists at these institutions who are just starting their careers (e.g. graduate students and postdoctoral fellows). Without such research we do not know whether young scientists at top universities are choosing not to persist because of the perceived impact the academic science career has on their current or projected family lives.

Our study is distinct in that it examines how graduate students, postdoctoral fellows, and faculty in leading science Ph.D. programs at 31 universities comprising 100 departments — programs that have a disproportionate impact in shaping the future of science and the next generation of scientists — view the impact of family factors on career and life satisfaction as well as projected career track.

## Methods

As part of the Perceptions of Women in Academic Scientist (PWAS) study, we selected a random sample of 3,455 scientists from the more than 14,000 graduate students, postdoctoral fellows, and tenure-track/tenured faculty members in the top 20 Ph.D. programs in all subfields of astronomy, physics and biology. Programs were ranked by the National Research Council (1995) and correlated with the rankings of *U.S. News* & *World Report* (2008). The PWAS survey ran from November 2008 through February 2009, using Web and phone surveys. The sample was stratified by rank in the career track and, where possible, we selected a disproportionately high sample of women within each rank. A personalized contact letter was initially sent to each of the potential respondents. It included a pre-incentive (cash that could be kept regardless of whether the respondent agreed to participate). Respondents were then contacted by a survey firm, Shulman, Ronca, and Buchavales, Inc. (SRBI), who emailed each potential respondent between eight and ten times. Each potential respondent received a unique ID with which to log into a Website and complete the survey. After the reminder emails, SRBI followed up with non-responders through a phone-calling effort, phoning them up to 20 times. Only 7.5 percent of the respondents completed the survey on the phone; 92 percent completed the Web-based survey. Overall, this combination of methods resulted in a high response rate of 72 percent or 2,503 respondents. This is a very high response rate for a survey of academics. For example, even the highly successful Carnegie Commission study of faculty resulted in only a 59.8 percent rate [8]. The final sample included 684 graduate students, 504 postdoctoral fellows, 446 assistant professors, 326 associate professors, and 543 full professors. This is an effective response of 75.3 percent among graduate students, 70.7 percent among postdoctoral fellows and 71.7 percent among faculty. An average of 81 persons from each university responded, roughly 25 from each department. A total of 1,300 biologists and 1,203 physicists responded to the study. (A complete guide to the survey questions is available upon request.)

For this paper, questions about significant influences on the pursuit of a science career, career experiences and impediments, and the work-family balance were analyzed from the PWAS survey. Some questions on the PWAS were replicated from existing studies of women in science, such as NSF ADVANCE surveys conducted by PIs examining gender differences in science at particular universities. We used two-tailed tests of statistical significance and logistic regression to analyze the data. T-tests are a premature measure of interpretation because they do not take into account any additional differences between men and women. Logistic regression allows estimates of how one variable predicts another variable, while adjusting for multiple other predictor variables. Thus, logistic regression permits comparison of men and women on key variables of interest while statistically equalizing their different demographic and human capital characteristics. Multivariate logistic regression analyses were weighted by rank, sex, and discipline to reflect chance of selection into the sample. Logistic regression coefficients are reported as odds ratios that center on the number 1. Values less than 1 have a negative effect on the dependent variables while values larger than 1 have a positive effect on the dependent variable. All analyses were conducted with the statistical program STATA version 9 from StataCorp, College Station, TX, USA.

### Ethics Statement

The Perceptions of Women in Academic Science received an expedited human subjects' approval 08/07/08 from Rice University's Institutional Review Board, which has been renewed every year of the three years of data collection (protocol number 09-008E). The PI requested and received permission that informed consent be waived for the survey portion of the study (data from which is reported in this paper). The letter that was sent requesting participation in the survey included an information sheet about the study as well as contact information for the PI and the director of the Rice University IRB should any complaints arise.

## Results

### Faculty

We found that work satisfaction, life satisfaction, and family factors were closely intertwined for faculty in the sciences. For example, survey analyses reveal that, when compared with their male colleagues, a greater proportion of women are dissatisfied in their roles as faculty members (15.5% of women vs. 11.5% of men, p = 0.0438, n = 1,302; Table 1). Yet there is no significant difference in the number of hours that women and men without children work per week (59.1 hours for women and 57.8 hours for men, p = 0.4219, n = 369). Both men and women with children work fewer hours than those without children, yet surprisingly, women with children do not work fewer hours than men with children (54.5 hours for women and 53.9 hours for men, p = 0.5742, n = 912).

**Table 1:** Descriptive Statistics for Key Independent Variables by Sex and Career Rank in PWAS Survey.

	Graduate Students		Postdoctoral Fellows		Faculty	
	Men	Women	Men	Women	Men	Women
Age	27.1	26.7	33.3	33.0	47.2	46.9
Married (%)	26.1	24.2	60.3	51.0	82.8	72.2***
Weekly hours worked	52.1	52.2	54.9	56.1	54.9	56.1
White (%)	65.8	63.8	59.3	60.9	83.4	81.8
Black (%)	1.2	3.4	0.3	0	0.7	0.9
Hispanic	7.5	4.3	6.0	6.4	2.5	3.2
Asian/Pacific Islander (%)	23.4	24.2	30.5	31.2	11.9	13.2
Foreign national (%)	33.8	32.4	61.4	65.2	39.9	35.0
Has children (%)	8.8	5.5	35.8	22.4	74.6	64.2***
Number of children	0.11	0.08	0.53	0.26	2.05	1.88*
Fewer children than desired (%)	20.3	39.4***	39.0	55.4***	24.5	45.4***
Overall dissatisfaction with life (%)	19.0	17.8	24.7	20.4	16.6	17.1
Income^1^	1.12	1.11	2.37	2.22	5.98	5.89
*Family-to-Work Spillover*						
Long work hours (%)	19.5	27.1*	19.2	23.8	11.6	18.5***
Balancing work and family (%)	23.7	27.9	31.8	33.2	32.0	47.6***
Overall dissatisfaction with present career stage (%)	17.9	17.1	15.6	16.2	11.5	15.5*
Concerned about not being able to have a family prior to graduate school(%)	7.2	28.5***	7.0	12.4*	2.5	12.1***
*Post-Graduate Plans*						
Seek tenure-track position (%)	66.5	60.1	84.0	69.2***	—	—
Seek teaching position (%)	33.5	41.3*	30.4	31.1	—	—
Seek research scientist position (%)	55.6	54.1	63.2	62.4	—	—
Seek industry position (%)	43.9	41.2	39.4	44.1	—	—
Seek job outside science (%)	25.2	26.4	16.4	20.3	—	—
*n*	*333*	*351*	*302*	*202*	*876*	*439*

* p<0.05.

** p<0.01.

*** p<0.001.

1 Is your university salary 1-below $40,000, 2-$40,000–49,999, 3-$50,000–$59,999, 4-$60,000–69,999, 5-$70,000–$79,999, 6-$80,000–89,999, 7-$90,000–99,999, 8-$100,000–109,999, 9-$110,000–119,999, 10-$120,000–129,999, 11-$130,000–139,999, 12-$140,000–149,999, 13-$150,000–159,999, 14-$160,000–169,999, 15-$170,000–179,999, 16-$180,000–189,999, 17-$190,000–199,999, 18-above $200,000.

Logistic regression was used to determine factors that influence the work satisfaction of scientists, as measured through their degree of agreement with the statement: “Overall, how satisfied are you with being a faculty member at your current institution?” Adjusting for sex, age, rank, income, marital status, number of children, weekly hours worked, scholarly productivity, and satisfaction with life outside work, we find that women are less satisfied than men. We also find that being more satisfied with life outside work positively predicts faculty satisfaction (Table 2, Col. 1). Those who are more satisfied with their lives are 60 percent more likely to report being satisfied with their careers. These findings hold even when accounting for whether faculty credit the science career with having fewer children than wished (Table 2, Col. 2).

**Table 2:** Logistic Regression of Faculty Satisfaction With Career and Life.^1^

	Satisfaction with Career		Satisfaction with LifeOutside Work	
Female	0.696**	0.707*	1.330*	1.540**
Age	0.981*	0.981*	1.026**	1.024**
Black	8.657**	8.713**	0.377***	0.465*
Hispanic	0.684	0.687	0.807	0.813
Asian/Pacific Islander	0.780	0.788	0.805	0.889
Assistant	0.695	0.701	1.302	1.394
Associate	0.743	0.748	1.067	1.105
Income	1.064***	1.064**	1.035	1.033
Married	0.960	0.962	1.601*	1.609*
Children (n)	1.051	1.046	1.141	1.078
Weekly hours worked (ln)	0.806	0.809	0.919	0.943
Publications (ln)	1.054	1.055	1.196	1.205
Life satisfaction	1.607***	1.600***	—	—
Career Satisfaction	—	—	1.612***	1.599***
Fewer children than wished	—	0.979	—	0.818***
n	1175	1175	1175	1175
R^2^	0.060	0.060	0.063	0.072

* p<0.05.

** p<0.01.

*** p<0.001.

1 −2 Very dissatisfied, −1 Somewhat dissatisfied, 0 Neither satisfied nor dissatisfied, 1 Somewhat satisfied, 2 Very Satisfied.

Despite the distinct differences between male and female faculty in satisfaction with work, there is no gender difference in life satisfaction for science faculty. In response to the question: “Overall, how satisfied are you with your life outside of work?” 17.1% of women vs. 16.6% of men (p = 0.9588, n = 1,295) reported being somewhat or strongly dissatisfied with their lives. Adjusting for sex, age, rank, income, marital status, number of children, weekly hours worked, scholarly productivity, and satisfaction with work, logistic regression analysis reveals that women, those who are older or married and those who are more satisfied with their careers have greater satisfaction with their lives outside of work (Table 2, Col. 3). Having fewer children than wanted due to the science career, however, has a significant negative effect on life satisfaction for faculty members, despite the fact that nearly twice as many women as men report having fewer children than desired because they pursued a science career (45.4% of women vs. 24.5% of men, p<0.0000, n = 1,302) and female faculty have fewer children than their male colleagues (1.2 children for women vs. 1.5 for men, p<0.0000, n = 1,297). That, once the aforementioned controls are included, (Table 2, Col. 4), women are actually *more* satisfied with their lives than men implies that having fewer children than wanted has a more pronounced effect on life satisfaction for male scientists.

### Graduate Students and Postdoctoral Fellows

Previous research shows that women are more likely than men to drop out of the pipeline before achieving tenure-track science faculty positions [1]. This makes it especially important to consider the impact (or beliefs about the impact) of the science career on family life among graduate students and postdoctoral fellows. Among graduate students and postdoctoral fellows, there is no gender difference in career satisfaction or number of children. A greater proportion of women, however, worry that a science career will keep them from having a family (for graduate students, 28.5% of women vs. 7.2% of men, p = 0.0000, n = 684; for postdoctoral fellows, 12.4% of women versus 7.0% of men, p = 0.0384, n = 504). It is not surprising then that by the time they reach the postdoctoral level, women are much *less* likely than men to report considering a tenure-track academic job at a research university (69.1% of women vs. 84.0% of men, p<0.0001, n = 472). But *both* men and women are *equally* likely to report considering a career outside science entirely (for graduate students, 25.2% of men and 26.4% of women, p = 0.7379, n = 639; for postdoctoral fellows, 16.4% of men and 20.3 of women, p = 0.2790, n = 463).

We used logistic regression analyses to determine factors that influence graduate students and postdoctoral fellows to consider choosing a career outside science (Table 3). We adjusted for sex, age, race, marital status, number of children, weekly hours worked, satisfaction with life outside work, and the fear that a science career would preclude having a family. None of these variables are significant. Indeed, having had fewer children than desired due to the science career is the only factor that predicts seeking a career outside science. Graduate students who have had fewer children than desired are 21% more likely to report considering a career outside science, and postdoctoral fellows are 29% more likely to report the same interest.

**Table 3:** Logistic Regression of Plans to Seek a Career Outside of Science.

	Graduate Students		Postdoctoral Fellows	
Female	1.204	1.042	1.257	1.251
Age	1.020	1.009	0.966	0.973
Black	2.230	2.265	—	—
Hispanic	0.577	0.456	0.841	0.899
Asian/Pacific Islander	1.138	1.342	1.073	1.139
Number of children	0.998	0.924	1.140	1.268
Married	1.005	1.062	0.667	0.588
Afraid can't have family due to science career	0.831	0.809	1.218	1.390
Life satisfaction	0.884	0.983	0.951	1.025
Fewer children than desired	—	1.210*	—	1.291*
*n*	621	411	446	395
R^2^	0.012	0.022	0.012	0.035

## Discussion

Female scientists at top universities not only have fewer children than their male colleagues but also are more likely to say that, due to the science career, they have fewer children than they want. Yet having fewer children than desired has a greater impact on *men's* life satisfaction—an important finding given that the relationship between pursuing a science career and men's family life is discussed little in the literature on gender and science. We also find that one in four graduate students and one in five postdoctoral fellows is considering a career outside science altogether. And those who say that the science career means they have fewer children than they want are more likely to desire a career outside science. Given these findings, universities would do well to re-evaluate how family-friendly their policies are. For example, top universities might leverage additional resources to help foster scientists' work-family balance, such as providing on-site day care. And our results reveal that mentoring programs—for both men and women—may need to focus more on how to balance academic science work with family life.
